# Trehalose Lipid Biosurfactant Reduces Adhesion of Microbial Pathogens to Polystyrene and Silicone Surfaces: An Experimental and Computational Approach

**DOI:** 10.3389/fmicb.2018.02441

**Published:** 2018-10-16

**Authors:** Tomasz Janek, Anna Krasowska, Żaneta Czyżnikowska, Marcin Łukaszewicz

**Affiliations:** ^1^Department of Biotechnology and Food Microbiology, Wrocław University of Environmental and Life Sciences, Wrocław, Poland; ^2^Department of Biotransformation, Faculty of Biotechnology, University of Wroclaw, Wroclaw, Poland; ^3^Department of Inorganic Chemistry, Wroclaw Medical University, Wroclaw, Poland

**Keywords:** biosurfactant, trehalose lipid, intermolecular interaction energy, adhesion, biofilm, microbial pathogens

## Abstract

*Rhodococcus fascians* BD8, isolated from Arctic soil, was found to produce biosurfactant when grown on n-hexadecane as the sole carbon source. The glycolipid product was identified as the trehalose lipid with a molecular mass of 848 g mol^−1^. The purified biosurfactant reduced the surface tension of water from 72 to 34 mN m^−1^. The critical micelle concentration of trehalose lipid was 0.140 mg mL^−1^. To examine its potential for biomedical applications, the antimicrobial and antiadhesive activity of the biosurfactant was evaluated against several pathogenic microorganisms. Trehalose lipid showed antimicrobial activity against resistant pathogens. The largest antimicrobial activities of trehalose lipid were observed against *Vibrio harveyi* and *Proteus vulgaris*. The highest concentration tested (0.5 mg mL^−1^) caused a partial (11–34%) inhibition of other Gram-positive and Gram-negative bacteria and 30% inhibition of *Candida albicans* growth. The trehalose lipid also showed significant antiadhesive properties against all of the tested microorganisms to polystyrene surface and silicone urethral catheters. The biosurfactant showed 95 and 70% antiadhesive activity against *C. albicans* and *Escherichia coli*, respectively. Finally, the role and application of trehalose lipid as an antiadhesive compound was investigated by the modification of the polystyrene and silicone surfaces. The intermolecular interaction energy calculations were performed for investigated complexes at the density functional level of theory. The results indicate that the presence of aromatic moieties can be substantial in the stabilization of trehalose lipid-surface complexes. The antimicrobial and antiadhesive activities of trehalose lipid make them promising alternatives to synthetic surfactants in a wide range of medical applications. Based on our findings, we propose that, because of its ability to inhibit microbial colonization of polystyrene and silicone surfaces, trehalose lipid can be used as a surface coating agent.

## Introduction

Drug resistance of bacterial and fungal infections is increasing worldwide. Microbial biofilm-associated infections are frequently refractory to conventional therapy because of resistance to antimicrobial agents (Sanchez et al., 2013; Malone et al., 2017). Approximately 80% of hospital-acquired infections are associated with the use of indwelling urinary catheters, where *Escherichia coli* and *Candida albicans* are among the most prevalent pathogens in urinary tract infections (Russo and Johnson, 2003; Patil et al., 2015). Biofilm-associated bacterial and fungal infections show uniform resistance to a wide spectrum of the currently available conventional agents, which implies that antimicrobial drugs against specifically targeted biofilm-associated infections are needed. Protection against pathogenic bacteria could be achieved by the preparation of new vaccines (Hotez et al., 2016) or searching for new antimicrobial compounds (Savoia, 2016) that inhibit adhesion of microorganisms to medical surfaces. Therefore, many laboratories are synthesizing or isolating new compounds that prevent the formation of biofilms or cause their elimination. Infectious diseases could be prevented by inhibiting biofilm formation using antiadhesive agents.

Biosurfactants are surface-active biomolecules that are produced by a variety of microorganisms (Biniarz et al., 2017). Microbial surfactants are considered to have some advantages over synthetic surfactants, such as low toxicity, high biodegradability, and specificity, and retention of physicochemical properties at multiple temperatures, salinities, and pH levels (De Almeida et al., 2016; Henkel et al., 2017). Because of their potential advantages, biosurfactants are widely used in many industries, such as chemistry, pharmaceutics, cosmetics, agriculture, and food production (Coutte et al., 2017; Perfumo et al., 2018).

Biosurfactants can be used as novel antibacterial and antiadhesive agents for medical applications. Previous research has highlighted the use of biosurfactants as antibiotic, antifungal, and antitumor agents (Janek et al., 2012, 2013a; Gudiña et al., 2013; Díaz De Rienzo et al., 2015). The antimicrobial activity of various kinds of biosurfactants against pathogenic microorganisms has been the subject of some thorough reviews and research publications (Díaz De Rienzo et al., 2016; Janek et al., 2016). A remarkable property of biosurfactants is their inhibitory activity against bacterial and fungal colonization of surfaces, including polystyrene, silicone (used in medical application, e.g., urethral catheters), and glass. Control of microbial growth is required in those biomedical fields where surfaces provide favorable conditions for the proliferation of microorganisms. In our previous work, the effect of biosurfactant pseudofactin on the adhesion and biofilm formation of Gram-positive and Gram-negative bacteria and yeast *C. albicans* was examined (Janek et al., 2012). Work by our group has demonstrated that the biosurfactant pseudofactin inhibits bacterial and *C. albicans* adhesion to polystyrene and silicone surfaces. Also, we have shown that biosurfactants have strong antiadhesive activities against bacterial and yeast strains on a polystyrene surface (Janek et al., 2013b). Vecino et al. (2018) have reported significant antiadhesive properties of glycolipopeptide cell-bound biosurfactants against all of the tested microorganisms, except *E. coli* and *C. albicans* (<30% antiadhesive activity). Although there are a few intensive studies on the antimicrobial and antiadhesive activities of biosurfactants, much work is still needed to understand the mechanism of action of the surface-active compounds.

The chemical and physical properties of some classes of biosurfactant are extensively studied, however, it's still very important to find the new biosurfactants and characterize their biological properties. Among biosurfactants, trehalose lipid represent a promising compounds due to their physicochemical and biological properties (Marqués et al., 2009; Franzetti et al., 2010). To date, there have been very few valuable studies concerning their biological activities. For example, the effect of succinoyl trehalose lipids on phosphatidylethanolamines and phosphatidylcholines was investigated (Zaragoza et al., 2009, 2010). Presented results got some insight into molecular interactions between the biosurfactant and the phospholipids of the membrane. As to their biological properties, succinoyl trehalose lipids have been found to possess hemolytic activity (Zaragoza et al., 2010). Besides, the same biosurfactant was less toxic than sodium dodecyl sulfate (SDS), and could be therefore used in cosmetic preparations (Marqués et al., 2009).

In this study, a trehalose lipid biosurfactant secreted by *Rhodococcus fascians* BD8 was employed to investigate its antimicrobial and antiadhesive activity against pathogenic bacteria. Since the effects of a surfactant might differ depending on the type of surface it adheres to; we tested its action on the adherence of the above pathogenic microorganisms to polystyrene, silicone, and glass surfaces. The non-covalent interactions between molecules of biological importance have been recognized as important in many processes in nature. An improved understanding of the stability and structural properties of biomolecules provides the detailed analysis of the strength of hydrogen bonds and London dispersion interactions (Hunter, 1993). Here we aimed to analyze the energetic and structural consequences of the interactions between trehalose lipid and medically-relevant surfaces, such as polystyrene and silicone. This type of analysis is scarcely available in the literature. To our knowledge, this is the first report of the antiadhesive efficacy of trehalose lipid, as evaluated by experimental and theoretical techniques.

## Materials and methods

### Strain and culture conditions

*Rhodococcus fascians* BD8 was isolated from a soil sample from the Arctic Archipelago of Svalbard (latitude 77°05'N, longitude 15°14′E). The strain was subcultured on Luria-Bertani agar plates (LB; 10 g L^−1^ of tryptone, 5 g L^−1^ of yeast extract, and 10 g L^−1^ of NaCl) for 24 h at 28°C. Next, the strain was preserved frozen at −80°C in the culture collection of the Laboratory of Biotransformation, University of Wroclaw, Poland.

The antimicrobial and antiadhesive activities of the trehalose lipid were tested on the following strains of microorganisms: (1) Gram-positive bacteria *Enterococcus hirae* ATCC 10542, *Enterococcus faecalis* JA/3 (clinical isolate, culture collection of the Wroclaw Medical University), *E. faecalis* ATCC 29212, and *Staphylococcus epidermidis* KCTC 1917; (2) Gram-negative bacteria *E. coli* 17-2 (clinical isolate, culture collection of the Wroclaw Medical University), *E. coli* ATCC 10536, *E. coli* ATCC 25922, *Proteus vulgaris* ATCC 27973, *Proteus mirabilis* ATCC 21100, and *Vibrio harveyi* ATCC 14126; (3) yeasts *C. albicans* ATCC 10231 and *C. albicans* SC5314. The bacteria were grown in LB medium at 37°C. The yeasts were grown in 6.7 g L^−1^ yeast nitrogen base (YNB, pH 5.5) broth (Difco Laboratories) containing 2% D-glucose at 37°C. The investigation of all three classes of microorganism allows a robust assessment of the antimicrobial and antiadhesive activities of the trehalose lipid.

### Biochemical and molecular characterization of the BD8 strain

In brief, BD8 was identified by Gram-staining and characterized biochemically using API Coryne at the species level according to the manufacturer's instructions (API Coryne, BioMerieux, France). Partial 16S rRNA gene sequencing analysis was used to characterize BD8 at the molecular level. Briefly, genomic DNA was isolated from a 1-day liquid culture using a GeneMATRIX Bacterial and Yeast Genomic DNA Purification kit 50 (EURx, Gdansk, Poland) and then the 16S rRNA gene was amplified by polymerase chain reaction (PCR) using primers 27F (5′-AGR GTT YGA TYM TGG CTC AG-3′) and 1492R (5′-GGT 96 TAC CTT GTT ACG ACT T-3′). The PCR products were sequenced and analyzed at the Institute of Biochemistry and Biophysics, Polish Academy of Sciences (Warsaw, Poland), using the standard shotgun sequencing reagents and a 454 GS FLX Titanium Sequencing System (Roche, Basel, Switzerland), according to the manufacturer's instructions. The gene sequences obtained from the BD2 isolate were compared in the National Center for Biotechnology Information (NCBI) for homology using BLAST and multiple-aligned with 16S rRNA gene sequences of different strains.

### Production, purification, and characterization of trehalose lipid biosurfactant

The BD8 strain was grown on Davis Minimal Media (DMM; 30 mmol L^−1^ K2HPO4, 14 mmol L^−1^ KH2PO4, 7.6 mmol L^−1^ (NH_4_)_2_SO_4_, 0.4 mmol L^−1^ MgSO_4_) supplemented with 20 g L^−1^ n-hexadecane at 28°C for 120 h. The culture sample (500 mL) was centrifuged at 8,000 × g at 4°C for 30 min, and the cell-free supernatant was extracted once with one volume of ethyl acetate and evaporated under vacuum. The crude biosurfactant was dissolved in methanol and purified via reversed-phase high-performance liquid chromatography (RP-HPLC, Waters, USA), equipped with an Xterra Prep RP18 OBD column (5 μm, 18 × 100 mm; Waters, USA). The solvent system consisted of solvent A (0.1% aqueous trifluoroacetic acid) and solvent B (0.1% trifluoroacetic acid in acetonitrile). The biosurfactant was eluted at a flow rate of 4 mL min^−1^ with a 38-min gradient (% A:B v/v): injection start (30:70) and 38 min (0:100).

The hemolytic activity of trehalose lipid (1 mg mL^−1^) was studied using blood agar plates. Plates were incubated at 37°C for 48 h. After the incubation time, the plates were inspected for zones of clearing around the wells. Phosphate-buffered saline (PBS, pH 7.4) was used as negative control and surfactin (Sigma-Aldrich) as a positive control. The assays were performed in three replicates.

The physicochemical properties of the purified biosurfactant, such as chemical structure, surface tension reduction (ST), and CMC, were evaluated following the protocols established in previous works (Janek et al., 2010). Therefore, the chemical structure of the biosurfactant was analyzed by matrix-assisted laser desorption/ionization time of flight mass spectrometry (MALDI-TOF MS); the surface tension was determined by a Tensiometer Kruss K100 (Kruss GmbH, Hamburg, Germany) at 25°C, according to the du Nouy's ring method (Huh and Mason, 1975); the mean particle size of trehalose lipid diluted in Milli-Q water was determined using a Zetasizer Nano-ZS (Malvern Instruments Ltd., Malvern, UK). The purified biosurfactant had a purity of >99%.

### Antimicrobial assay

The microdilution method was used to determine the antimicrobial activity of the trehalose lipid. The experiments were performed in 96-well, flat-bottomed plastic microplates (Sarstedt, Nümbrecht, Germany). Portions of 50 μL of an appropriate medium (LB broth for bacteria and YNB for yeast) containing different concentrations of trehalose lipid were dispensed into the wells of the microplate. Then, 50 μL of bacterial and yeasts suspension (6 log CFU mL^−1^) were added to each well, providing final concentrations of trehalose lipid ranging from 0.035 to 0.5 mg mL^−1^. Each plate included controls without trehalose lipid. The systems were incubated at 37°C for 24 h, and after the incubation period, the growth of microorganisms was monitored by measuring optical density at 600 nm using an Asys UVM 340 (Biogenet) microplate reader. The growth inhibition percentages at different biosurfactant concentrations for all tested microorganisms were calculated following Equation (1):

(1) $$\begin{array}{r} \%\;Growth\;inhibition=\left[1-\frac{OD_{c}}{OD_{0}}\right]\times 100 \end{array}$$

where *OD*_c_ represents the optical density of the well with a given trehalose lipid concentration and *OD*_0_ is the optical density of the control well (without trehalose lipid). Assays were carried out as three biological replicates, each in triplicate. The results are presented as means ± standard deviation (SD).

### Anti-adhesion assays

The antiadhesive activity of trehalose lipid was tested against the same microorganisms described in the antimicrobial assay. Several trehalose lipid concentrations were tested ranging from 0.035 to 0.5 mg mL^−1^. The wells of a sterile 96-well, flat-bottomed polystyrene plate were filled with 100 μL of trehalose lipid biosurfactant solution dissolved in PBS (pH 7.4) following the methodology reported elsewhere (Janek et al., 2012). The plates were incubated for 2 h at 37°C on a rotary shaker (MixMate, Eppendorf, Hamburg, Germany) at 300 rpm and subsequently washed twice with PBS. Control wells contained only PBS. Negative control wells contained trehalose lipid (0.5 mg mL^−1^) while positive control wells contained only PBS. A 100-μL aliquot of a washed microbial suspension in PBS (pH 7.4), adjusted to an optical density of 1.0 for bacterial and 0.6 for *C. albicans* strains, was added to each well and incubated for 2 h at 37°C. Unattached cells were removed by washing the wells three times with PBS. Subsequently, the plates were stained with 0.1% crystal-violet for 5 min and again washed three times with PBS. The dye bound to the adherent microorganisms was resolubilized with 150 μL of isopropanol-0.04 N HCl and 50 μL of 0.25% SDS per well, and the optical density was measured at 590 nm. Assays were carried out three times, each consisting of three technical replicates. The results are presented as means ± SD.

### Biofilm formation assay

To generate *E. coli, E. faecalis, E. hirae*, and *C. albicans* biofilms on Thermanox polystyrene coverslips (Nalgen Nunc International Co., Rochester, NY) and glass microscopic coverslips (Menzel-Glaser, Germany), 10 μL of overnight cultures (10^8^ CFU mL^−1^) of *E. coli* ATCC 25922, *E. faecalis* ATCC 29212, and *E. hirae* ATCC 10541 were added into 1,000 μL of fresh LB medium, and the same volume of *C. albicans* SC5314 was added into 1,000 μL of fresh RPMI-1640 medium, with and without trehalose lipid biosurfactant (0.25 mg mL^−1^). The 24-well plate (Nalgen Nunc International Co., Rochester, NY) was incubated in an orbital incubator (100 rpm) at 37°C for 24 h. To remove unattached cells, the polystyrene and glass coverslips were removed and rinsed twice with 1 mL of the PBS solution. Removed polystyrene glass slides were stained for 30 min at 37°C with 1 mL of 0.6% Live/Dead BacLight viability stain (Molecular Probes, Eugene, OR) dissolved in PBS, and PBS-containing concanavalin A-Alexa Fluor 488 (Molecular Probes, Eugene, OR) conjugate (0.025 mg mL^−1^) for *C. albicans* biofilms. The confocal laser scanning microscopy (CLSM) observations were carried out using an Olympus FluoView 500 (Olympus Optical Co. Ltd., Japan) microscope.

Biofilm formation in urethral catheters was carried out under dynamic conditions using a peristaltic pump, where the flow of culture with or without trehalose lipid trough urethral catheters was 50 mL h^−1^. The catheters were incubated at 37°C overnight as previously described (Janek et al., 2012). Next, the catheters were washed with PBS. The catheters were stained with 0.1% crystal-violet for 20 min, again washed three times with PBS, and allowed to dry at room temperature for 15 min before the examination. In a parallel experiment, the catheters were pretreated with trehalose lipid (0.25 mg mL^−1^) dissolved in PBS, incubated for 2 h at 37°C, and subsequently washed twice with PBS. Assays were carried out three times.

### Total intermolecular interaction energy calculations

The geometry of complexes was optimized at the B3LYP/6-31G(d,p) level of theory using the Gaussian03 package (Frisch et al., 2009). Optimization was performed with inclusion of solvent effects (water) using a polarizable continuum model (PCM) (Cancès et al., 1997; Tomasi et al., 1999, 2005). The total intermolecular interaction energy was calculated using the supermolecular approach and corrected for the basis set superposition error (BSSE) (Boys and Bernardi, 1970). The result was defined as the electronic energy difference between the dimer and the energies of isolated monomers. Cramer and Truhlar (2009) proposed many exchange-correlation functionals, which can in the proper way characterize the non-covalent interactions between the molecules of biological importance. Later, Czyznikowska and Bartkowiak (2011) demonstrated that, in the case of weak non-bonding and stacking interactions, the best agreement of M06 functional results with those obtained with the aid of reference method. Therefore, in the present study, the interaction energy was computed at the M06/6-31G(d,p) level of theory. The molecular electrostatic potential on the electron isodensity surfaces (0.001 a.u.) of investigated monomers based on data obtained at the same level of theory (Bulat et al., 2010) in the gas phase.

### Cytotoxicity assay

Normal Human Epidermal Keratinocytes (NHEK) (PromoCell GmbH, Heidelberg, Germany) were cultured in Keratinocyte growth medium (KGM-Gold^TM^ from Lonza, Basel, Switzerland), supplemented with 10% fetal bovine serum (FBS), and antibiotics (10 U mL^−1^ penicillin and 10 μg mL^−1^ streptomycin) at 37 °C, with 5% CO_2_. For all the experiments, the NHEK cells were used at 80% confluence following 2–6 passages.

Cytotoxicity was measured by MTT (3-(4,5-dimethylthiazol-2-yl)-2,5-diphenyltetrazolium bromide) (Sigma, St Louis, MO, USA) cell proliferation assay (Mosmann, 1983). For these experiments, NHEK cells were plated in 96 well plates (1 × 10^4^ cells per well) and were allowed to attach for 24 h. Next, the cells were incubated for 24 and 48 h with increasing concentrations of trehalose lipid (0–0.5 mg mL^−1^). After incubation, cell viability was assessed by incubating the cells with 0.5 mg mL^−1^ of MTT for another 4 h. Then, the supernatants were removed from the plates and the precipitated formazan was dissolved in 50 mL of DMSO. The absorbance of the resulting solutions was read at a wavelength of 550 nm. The results were expressed as mean ± SD. Statistical significance was determined using Student's *t*-test. The significance level was set at *P* < 0.05. The 50% inhibitory concentration (IC_50_) values (mg mL^−1^) were defined as the compound concentrations reducing absorbance to 50% of control values.

## Results and discussion

### Identification of StrainBD8 and characterization of biosurfactant

**Figure 1:**
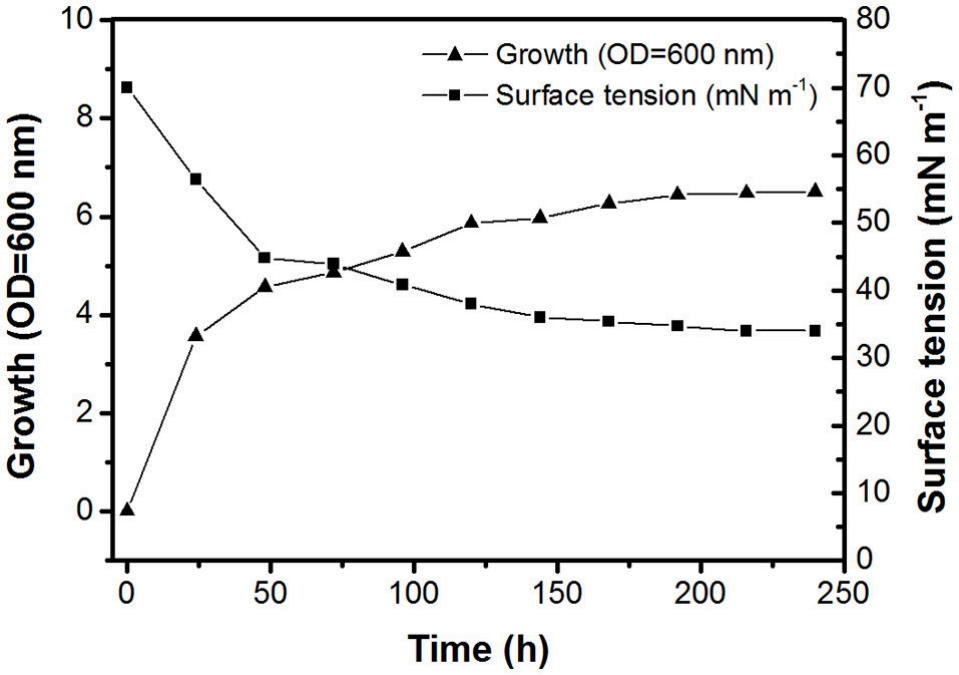
Time course of biosurfactant production, cell growth, and surface tension of *R. fascians* BD8 grown on mineral salt medium with 20 g L^−1^ n-hexadecane at 28°C.

The BD8 strain isolated from Arctic soil was Gram-positive, aerobic, and pleiomorphic. The optimal growth temperature for the BD8 strain in DMM with n-hexadecane was 28°C. The API Coryne strip returned positive results for pyrazinamidase, α-glucosidase, and glucose fermentation. The following were negative on the strip: nitrate reduction, gelatin hydrolysis, pyrrolidonyl arylamidase, alkaline phosphatase, β-glucuronidase, β-galactosidase, nacetyl-β-D-glucosaminidase, urease, and fermentation of ribose, xylose, mannitol, lactose, sucrose, and glycogen. On the basis of the morphological and metabolic pattern obtained, strain BD8 showed high similarity to a type strain of *R. fascians*. Comparison of the 16S rRNA nucleotide sequence from strain BD8 (GenBank accession number MH915580) with sequences in the GenBank database was performed using the online BLAST program. Because the gene sequence comparison demonstrated 99% similarity to *R. fascians* KX380901, the identity of the BD8 strain was confirmed. The growth and production of biosurfactant were observed when 20 g L^−1^ n-hexadecane was used as a carbon source. Figure 1 shows the growth and surface activity of *R. fascians* BD8, up to 240 h. It was observed that maximum decrease in the surface tension (in the range of 36–34 mN m^−1^) was achieved within 120 h of onset of the fermentation, which was not reduced further even after 240 h. The crude biosurfactants were extracted with ethyl acetate from a culture of the BD8 strain, as described in the Materials and Methods section. The surface-active compounds obtained by chemical extraction were subjected to RP-HPLC. With a linear gradient of acetonitrile and 0.1% aqueous trifluoroacetic acid, the compounds were resolved into twelve fractions (Supplementary Figure S1). Several fractions of compounds obtained after purification by HPLC from *R. fascians* BD8 were used. In all the stages of method development, the surface tension action was exclusively displayed by the fraction at a retention time of 24.2 min, while the other fractions did not show any surface tension activity. The surface tension of the major fraction (retention time: 24.2 min) was found to be lowest (i.e., 34 mN m^−1^).

Structural characterization of the purified biosurfactant can also be carried out using numerous techniques (Biniarz et al., 2017). MS provides the best method for characterization of biosurfactants. Electrospray ionization mass spectrometry (ESI-MS) (Luong et al., 2018), and more recently MALDI-TOF MS (Janek et al., 2010; Kügler et al., 2014), have been used for the characterization of the biosurfactant structures. Previous studies reveal that the surface-active compounds produced by *Rhodococcus* sp. are trehalose lipids (Franzetti et al., 2010; Sambles and White, 2015). This was confirmed here by MALDI-TOF MS analysis. The mass spectrum and chemical structure of the biosurfactant are illustrated in Supplementary Figure S2. The peak of m/z 871.8 for [M+Na]^+^ is the molecular ion of trehalose lipid, which gave rise to fragments of 709.8 and 277.8 m/z. Consequently, the confirmed structure of the biosurfactant secreted by *R. fascians* BD8 is shown in Supplementary Figure S2. The trehalose lipid produced by *R. fascians* BD8 differs from most rhodococci-produced trehalose lipids as they only carry one branched acyl chain (C16/C17), compared to the previously described double-acyl chains analogs of mycolic and succinoyl trehalose lipids (Ueda et al., 2001; Marqués et al., 2009).

**Figure 2:**
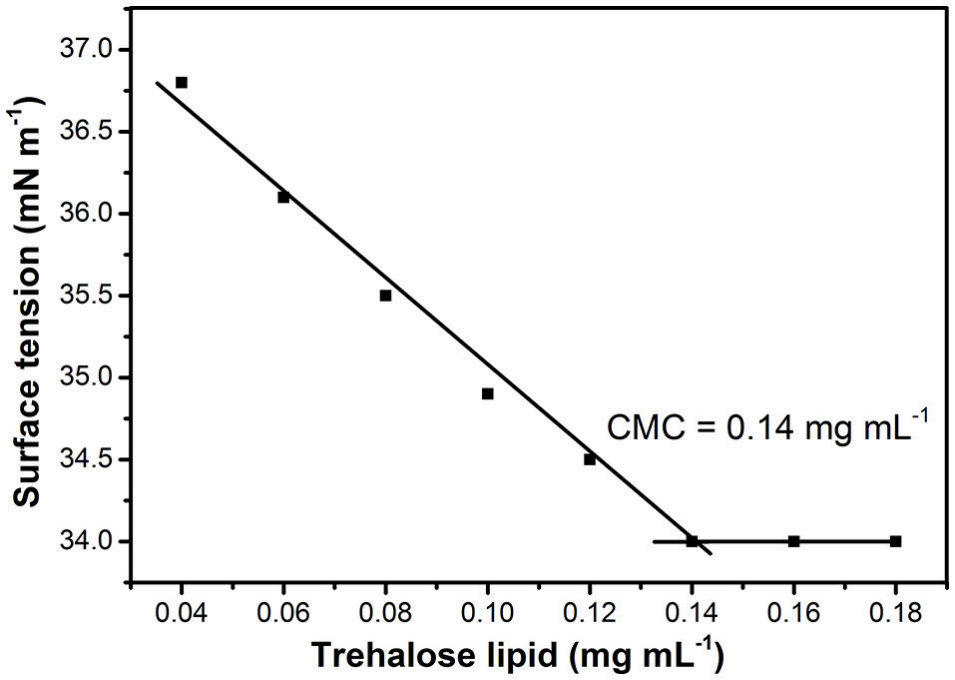
Effect of trehalose lipid concentration on surface tension. The CMC was determined from the intersection of regression lines that describe two parts of the curve, below and above the CMC.

Their potential to reduce the surface tension of liquids is an important property of microbial surfactants. Figure 2 shows that the surface tension decreased with the increase of glycolipid concentration. The trehalose lipid reduced surface tension of the water from 72 to 34 mN m^−1^. The CMC, determined with the aid of a series of concentrations, was around 0.140 mg mL^−1^ (Figure 2), which is in agreement with previous studies concerning biosurfactants isolated from other *Rhodococcus* spp. strains (Kuyukina et al., 2001; Mutalik et al., 2008). Knowledge of the aggregation behavior is a vital part of understanding how trehalose lipids participate as components in micelle systems. The size variation of the hydrodynamic diameter was evaluated by determination of dynamic light scattering (DLS) at various concentrations of trehalose lipid (Table 1). The attained results show that in concentrations greater than the CMC point, the aggregate size was increased from 60 to 157 nm as the trehalose lipid concentration was increased from 0.15 to 0.5 mg mL^−1^. Altogether, these results indicate that the trehalose lipid undergoes a micelle to vesicle transition in aqueous solution by increasing its concentration. The relatively large hydrodynamic diameter of the micelles at concentrations >3 × CMC suggests that these structures might be cylindrical, rather than spherical.

**Table 1:** The effect of trehalose lipid concentration on aggregate size.

**Biosurfactant**	**Concentration (mg mL^−1^)**	**Hydrodynamic diameters (nm)**	**Polydispersity index (PDI)**
Trehalose lipid	0.15	60.8 ± 1.4	0.137 ± 0.04
	0.20	71.7 ± 1.7	0.129 ± 0.11
	0.30	81.8 ± 0.9	0.181 ± 0.09
	0.40	150.0 ± 1.4	0.361 ± 0.12
	0.50	157.0 ± 2.1	0.266 ± 0.08

*The results represent the average of triplicate experiments ± SD*.

Our structural and physicochemical analyses of the trehalose lipid secreted by *R. fascians* BD8 identified an amphiphilic character, which could be relevant for their use in industrial-scale production and applications. On the other hand, it was concluded that the trehalose lipid produced by *R. fascians* BD8 did not exhibit hemolytic activity. These results did not correlate with the hemolytic properties of succinoyl trehalose lipid produced by *Rhodococcus* sp. (Zaragoza et al., 2010), suggesting that this trehalose lipid could be a promising candidate for medical applications.

### Antimicrobial activity of the trehalose lipid

The trehalose lipid biosurfactant was screened for antimicrobial efficacy against both Gram-positive and Gram-negative bacterial strains, as well as against *C. albicans*. We found that the trehalose lipid (0.5 mg mL^−1^) caused growth inhibition of *P. vulgaris* ATCC 27973 and *V. harveyi* ATCC 14126. Besides, Table 2 shows a remarkable low growth inhibition for *E. faecalis* ATCC 29212, *S. epidermidis* KCTC 1917, *E. coli* ATCC 25922, and *C. albicans* SC5314. The presented results indicate that the isolated trehalose lipid showed a broad range of potential antimicrobial activity against all tested bacterial and fungal pathogenic strains. Also, in the present study the activity of trehalose lipid toward Gram-positive and Gram-negative bacterial strains is known to have different effects. Similar results were presented by Vollbrecht et al. (1999). They revealed that trehalose lipid secreted by *Tsukamurella* sp. strain DSM 44370 exhibit activity against Gram-positive bacteria, while the Gram-negative bacteria were not sensitive.

**Table 2:** Antimicrobial activity of the trehalose lipid produced by *R. fascians* BD8 against pathogenic microorganisms.

**Microorganisms**	**Growth inhibition (%)**					
	**Trehalose lipid concentration (mg mL**^**−1**^**)**					
	0.500	0.250	0.200	0.150	0.075	0.035
*Enterococcus hirae* ATCC 10541	32 ± 0.27	31 ± 0.17	27 ± 0.24	27 ± 0.07	12 ± 0.32	4 ± 0.04
*Enterococcus faecalis* JA/3	34 ± 0.14	26 ± 0.21	21 ± 0.51	14 ± 0.07	4 ± 0.09	3 ± 0.07
*Enterococcus faecalis* ATCC 29212	15 ± 0.03	13 ± 0.17	12 ± 0.09	6 ± 0.07	5 ± 0.06	0 ± 0.28
*Staphylococcus epidermidis* KCTC 1917	14 ± 0.03	10 ± 0.12	8 ± 0.37	4 ± 0.07	2 ± 0.47	2 ± 0.23
*Escherichia coli* 17-2	25 ± 0.09	24 ± 0.12	20 ± 0.23	6 ± 0.07	4 ± 0.04	3 ± 0.21
*Escherichia coli* ATCC 10536	25 ± 0.37	21 ± 0.13	18 ± 0.19	10 ± 0.17	49 ± 0.23	3 ± 0.57
*Escherichia coli* ATCC 25922	11 ± 0.21	9 ± 0.17	6 ± 0.24	5 ± 0.11	2 ± 0.23	2 ± 0.31
*Proteus vulgaris* ATCC 27973	89 ± 0.32	61 ± 0.12	48 ± 0.14	37 ± 0.51	22 ± 0.47	9 ± 0.17
*Proteus mirabilis* ATCC 21100	27 ± 0.21	25 ± 0.15	19 ± 0.02	17 ± 0.07	11 ± 0.05	0 ± 0.29
*Vibrio harveyi* ATCC 14126	95 ± 0.47	72 ± 0.13	54 ± 0.09	33 ± 0.07	21 ± 0.23	11 ± 0.04
*Candida albicans* ATCC 10231	30 ± 0.47	27 ± 0.31	19 ± 0.08	14 ± 0.07	10 ± 0.03	9 ± 0.27
*Candida albicans* SC5314	7 ± 0.53	7 ± 0.37	5 ± 0.17	3 ± 0.07	1 ± 0.47	0 ± 0.41

*The results represent the average of triplicate experiments ± SD*.

Lipopeptide and glycolipid biosurfactants isolated from microorganisms have inhibitory activities against various species of Gram-positive and Gram-negative bacteria, as well as fungi (Inès and Dhouha, 2015; Meena and Kanwar, 2015). Biosurfactants behave similarly to synthetic surfactants, and their proposed mechanism of action consists of intercalation into biological membranes and destruction by their permeabilizing effect, leading to cell death (Sotirova et al., 2008). For instance, fengycin and surfactin homologs have a wide antibacterial spectrum against many Gram-positive bacteria, including against *Micrococcus luteus, E. coli*, and *Aspergillus niger* (Sun et al., 2006). In contrast to lipopeptides and rhamnolipids (Mnif and Ghribi, 2015; Elshikh et al., 2017), the trehalose lipid shows a much weaker dose-dependent antimicrobial activity against Gram-positive and Gram-negative bacterial strains. A previous study of the antibacterial effect of natural microbial products against several pathogenic bacteria and fungi indicated that the antibacterial activities of trehalose lipids (Inès and Dhouha, 2015) are different from the properties of another biosurfactant. However, in this study, we found that the trehalose lipid isolated from *R. fascians* BD8 had specific antagonistic activity against Gram-negative bacteria, especially *P. vulgaris* and *V. harveyi*.

### Antiadhesive activity

Adhesion of pathogenic microorganisms to solid surfaces or infection sites has been found to be inhibited by biosurfactants capable of modifying the physicochemical properties of the surface, thereby reducing adhesion and biofilm formation on a given biomaterial (Janek et al., 2012). Biosurfactants as antimicrobial agents might inhibit bacterial adhesion to surfaces. However, there is no information on the antiadhesive activity of the trehalose lipids under investigation. Therefore, the trehalose lipid was studied for its antiadhesive activity against Gram-positive and Gram-negative bacterial strains, as well as fungal strains, such as *C. albicans*. The pretreatment of polystyrene surfaces with trehalose lipid significantly decreased the adhesion of all tested microorganisms. The results of the antiadhesive activity for the trehalose lipid are shown in Table 3, which suggests that this compound exhibited good antiadhesive activity and this effect was concentration-dependent. As can be seen, the antiadhesive activity strongly depends on the type of microorganisms. The trehalose lipid reduced the adhesion to polystyrene for *P. mirabilis, E. coli, E. hirae*, and *C. albicans* by 70–95%, while for *S. epidermidis, E. faecalis*, and *P. vulgaris* adhesion to polystyrene was reduced by 41–44% using 0.5 mg mL^−1^ trehalose lipid.

**Table 3:** Antiadhesive activity of the trehalose lipid produced by *R. fascians* BD8 against pathogenic microorganisms.

**Microorganism**	**Microbial adhesion inhibition (%)**							
	**Trehalose lipid concentration (mg mL**^**−1**^**)**							**Control (PBS)**
	0.500	0.350	0.250	0.200	0.150	0.075	0.035	0
*Enterococcus hirae* ATCC 10541	70 ± 0.13	71 ± 0.07	71 ± 0.07	67 ± 0.13	66 ± 0.13	61 ± 0.20	58 ± 0.13	0
*Enterococcus faecalis JA/3*	43 ± 0.13	41 ± 0.20	35 ± 0.13	32 ± 0.20	27 ± 0.13	19 ± 0.07	18 ± 0.07	0
*Enterococcus faecalis* ATCC 29212	60 ± 0.20	58 ± 0.13	48 ± 0.20	47 ± 0.13	41 ± 0.13	32 ± 0.13	24 ± 0.13	0
*Staphylococcus epidermidis* KCTC 1917	41 ± 0.13	35 ± 0.13	33 ± 0.13	31 ± 0.13	28 ± 0.13	24 ± 0.13	10 ± 0.13	0
*Escherichia coli* 17-2	62 ± 0.20	61 ± 0.33	56 ± 0.07	45 ± 0.07	41 ± 0.07	34 ± 0.33	24 ± 0.13	0
*Escherichia coli* ATCC 10536	70 ± 0.20	64 ± 0.20	54 ± 0.13	52 ± 0.07	48 ± 0.20	35 ± 0.13	21 ± 0.13	0
*Escherichia coli* ATCC 25922	67 ± 0.33	61 ± 0.07	53 ± 0.33	51 ± 0.07	44 ± 0.07	38 ± 0.07	23 ± 0.33	0
*Proteus vulgaris* ATCC 27973	44 ± 0.20	38 ± 0.20	31 ± 0.07	26 ± 0.07	22 ± 0.13	19 ± 0.07	14 ± 0.33	0
*Proteus mirabilis* ATCC 21100	69 ± 0.20	64 ± 0.20	56 ± 0.13	43 ± 0.13	41 ± 0.13	37 ± 0.20	30 ± 0.07	0
*Vibrio harveyi* ATCC 14126	50 ± 0.20	44 ± 0.07	35 ± 0.13	33 ± 0.20	28 ± 0.20	25 ± 0.20	19 ± 0.13	0
*Candida albicans* ATCC 10231	95 ± 0.07	89 ± 0.13	85 ± 0.20	77 ± 0.20	71 ± 0.13	52 ± 0.20	49 ± 0.20	0
*Candida albicans* SC5314	90 ± 0.07	87 ± 0.13	84 ± 0.07	76 ± 0.13	71 ± 0.20	62 ± 0.07	53 ± 0.20	0

*PBS was used as control and set at 0% as no microbial inhibition occurs. Values ± confidence interval, n = 9*.

Our results are in accordance with those of Vecino et al. (2018) who reported that the pre-treatment of a polystyrene surface with biosurfactants produced by *Lactobacillus pentosus* and *Lactobacillus paracasei* inhibited bacterial and *C. albicans* adhesion by 30–81%. In another study, Gudiña et al. (2010b) reported that the highest antiadhesive activity of the biosurfactant produced by *L. paracasei* was observed against *Staphylococcus aureus* (72.0%) and *S. epidermidis* (62.1%) at a concentration of 25 mg mL^−1^. Furthermore, the *L. paracasei* ssp. *paracasei* A20 strain produces biosurfactant that exhibits antiadhesive activity against *Lactobacillus reuteri* strains (78%) and *Lactobacillus casei* strains (56–63%) at a biosurfactant concentration of 50 mg mL^−1^ (Gudiña et al., 2010a).

Usually, the effect of surfactant on the adhesion of undesirable microorganisms is attributed to modifications in the surface properties. However, in most cases, the precise mechanisms of such activity have not been fully explained, and more complex mechanisms can be involved. Therefore, we suggested a novel application of trehalose lipid as an antiadhesive agent. The efficiency of trehalose lipid against the adhesion of microorganisms, even at low concentrations, makes it a potential surface active compound for therapeutic applications.

### Trehalose lipid reduces biofilm formation on polystyrene, glass, and silicone

**Figure 3:**
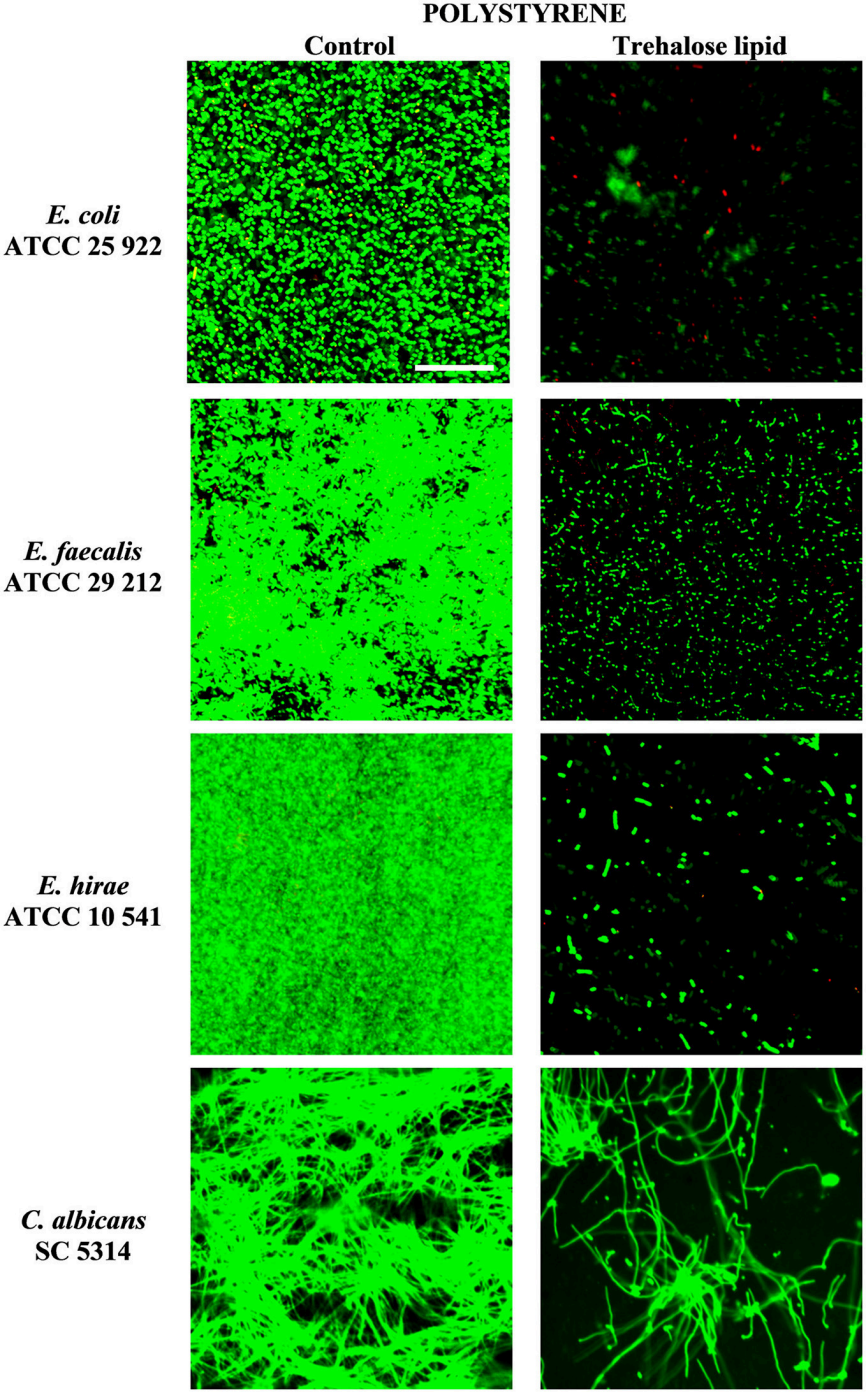
Confocal scanning laser microscopy images of biofilm formation on polystyrene coverslips by various bacterial and *C. albicans* strains.

**Figure 4:**
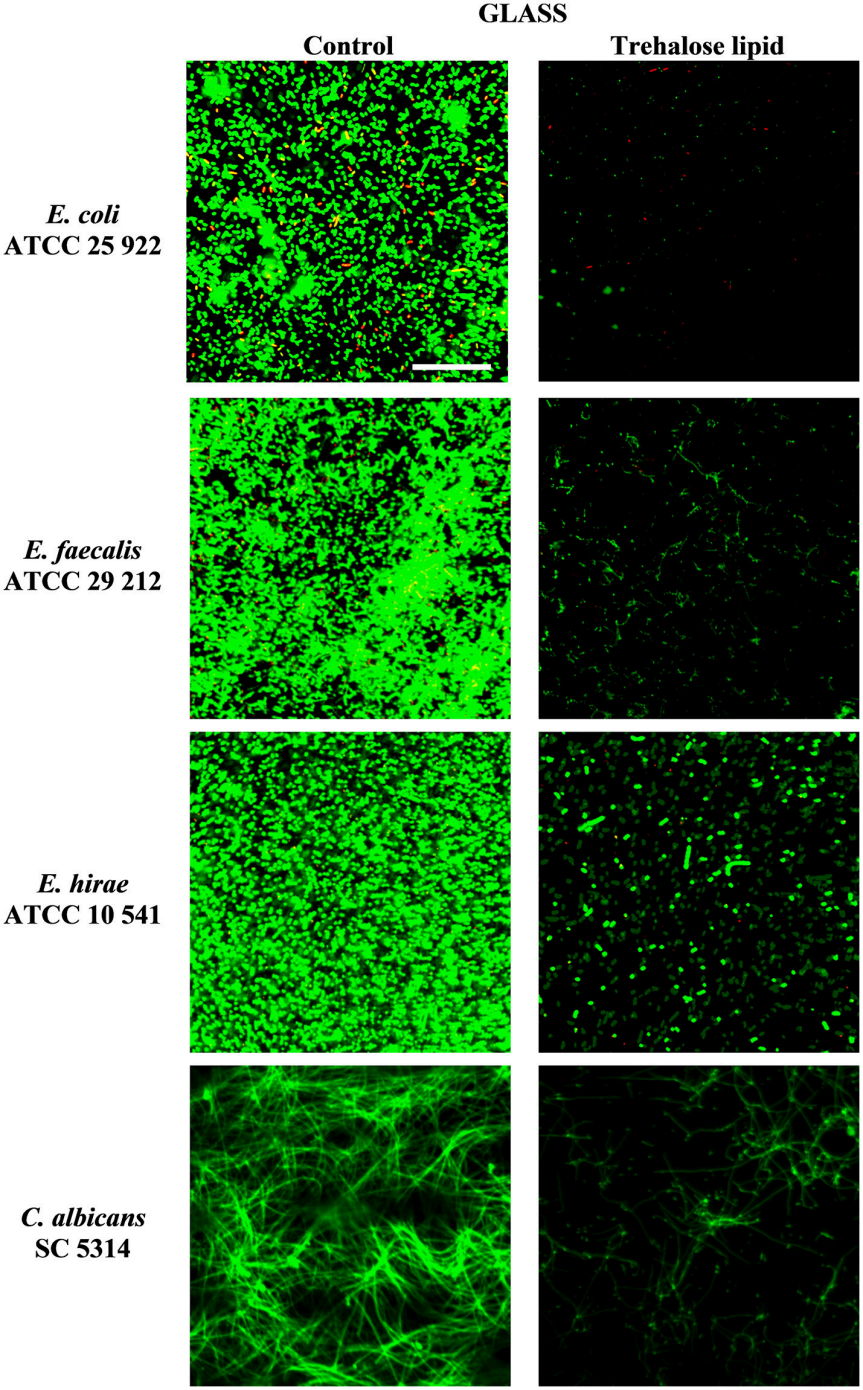
Confocal scanning laser microscopy images of biofilm formation on glass microscopic coverslips by various bacterial and *C. albicans* strains.

The inhibition of biofilm formation is due to the capacity of biosurfactants to modify the physicochemical properties of the surface to reduce adhesion and biofilm formation. Therefore, the potential effect of biosurfactants on biofilm inhibition could be the result of the interaction between the negatively and positively charged parts of polystyrene and silicone surfaces and carbohydrates regions of trehalose lipid molecules. Thus, we have tested the influence of trehalose lipid on biofilm formation on different materials. Visualization with the Live/Dead BacLight staining and concanavalin A-Alexa Fluor 488 with CLSM showed a reduction of bacterial and *C. albicans* biofilms in the presence of trehalose lipid (final concentration 0.25 mg mL^−1^) in the culture medium. The growth of *E. coli, E. faecalis, E. hirae*, and *C. albicans* biofilms on polystyrene and glass are shown in Figures 3, 4. In the control group, most of the colonies and microbes stain green, which indicates that the microorganisms were viable and formed a biofilm. However, in the presence of trehalose lipids, fewer colonies of bacteria and *C. albicans* were seen. This result is in accordance with the results obtained from the antiadhesive activity.

**Figure 5:**
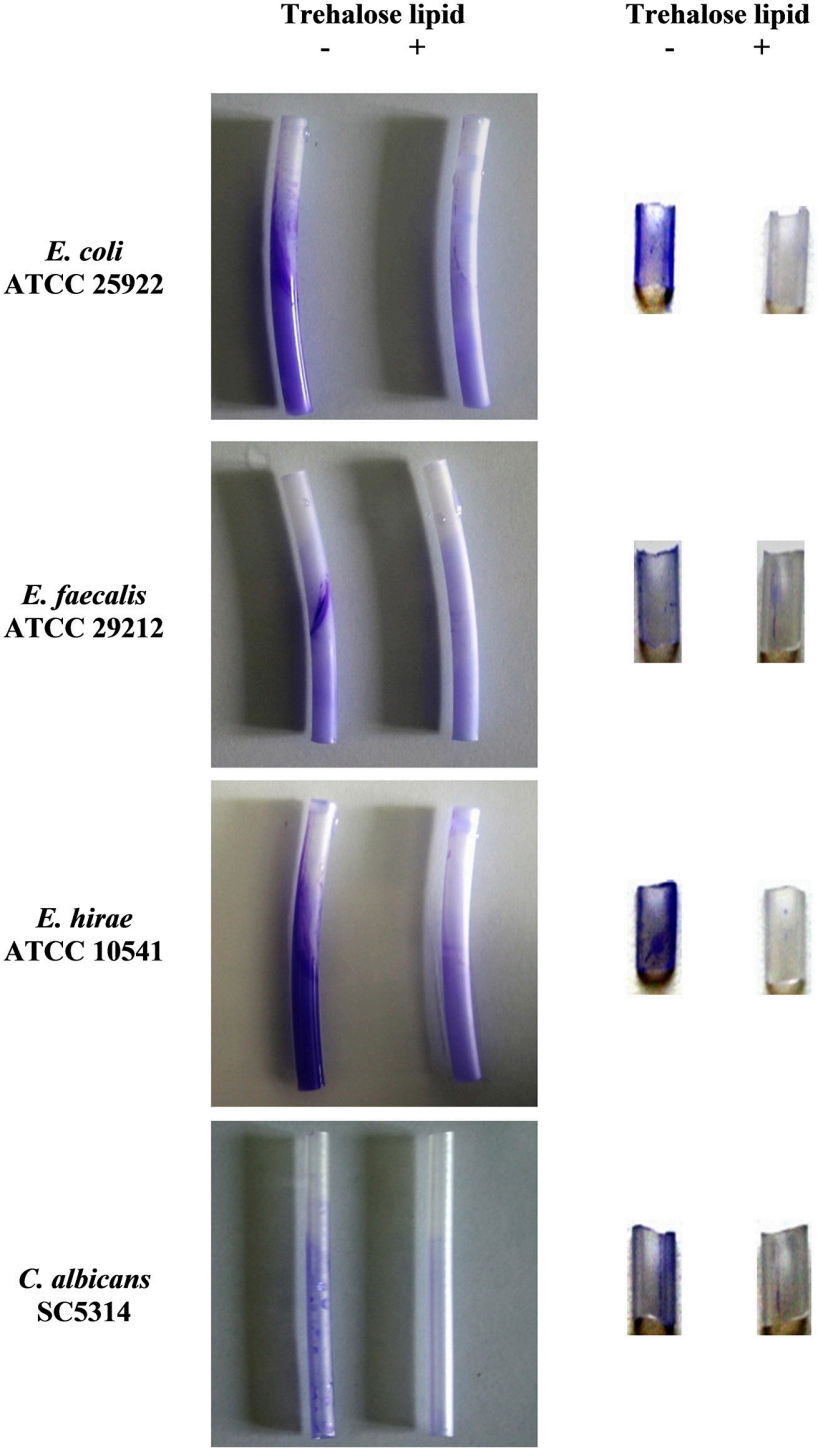
Trehalose lipid inhibits biofilm formation on silicone urethral catheters. The sterile urethral catheters were incubated with microorganisms containing 0.25 mg mL^−1^ trehalose lipid **(Left)**; the urethral catheters were pre-incubated with 0.25 mg mL^−1^ trehalose lipid **(Right)**. Biofilms were visualized by staining with crystal violet.

Silicone, as a medical material, is widely used due to its mechanical properties and biocompatibility. However, silicone is susceptible to microbial colonization, and a biofilm can form rapidly on the surfaces. Therefore, we evaluated the effect of trehalose lipid against *E. coli, E. faecalis, E. hirae*, and *C. albicans* biofilm formation on silicone. Our results demonstrated the ability of the trehalose lipid to inhibit the adhesion and biofilms formation (Figure 5). The pretreatment of silicone urethral catheters with trehalose lipid prior to inoculation with medium was as effective as including the surface active compound in the growth medium. In previous studies, the initial adhesion of bacteria and *C. albicans* to silicone was inhibited by pseudofactin II biosurfactant (Janek et al., 2012). Another report has shown that biosurfactants from *L. casei* and *Pseudomonas aeruginosa* inhibit/disperse biofilms (Chebbi et al., 2017; Merghni et al., 2017). Biosurfactants can reduce the hydrophobicity of the substratum surface, and then interfere with the processes of microbial adhesion and desorption, as explained below.

### Intermolecular interactions

**Figure 6:**
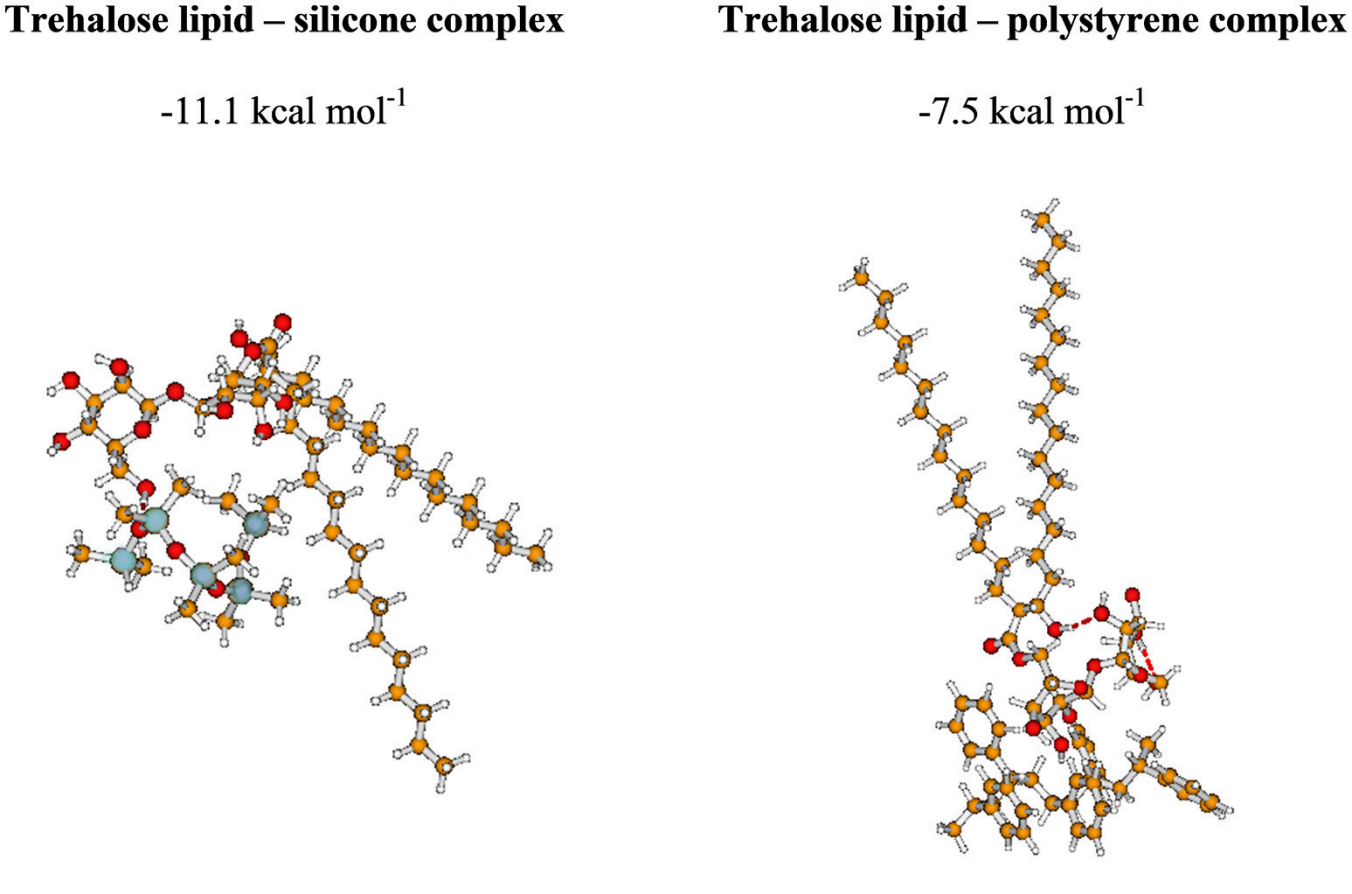
The most stable complexes investigated in the present study. BSSE-corrected intermolecular interaction energy (given in kcal mol^−1^).

Although the properties of surfactants have been extensively studied, to our knowledge, there is still a dearth of data concerning their intermolecular interactions. In this section, we present our preliminary results aimed at gaining an insight into the interactions between the trehalose lipid and surfaces. Because the computational cost rapidly increases with the number of basic functions, here we report the results describing interactions between one molecule of surfactant and fragments of polystyrene and silicone surfaces containing four and five molecules, respectively. The structural and energetic properties of the investigated complexes are presented in Figure 6 and Supplementary Movies 1, 2.

**Figure 7:**
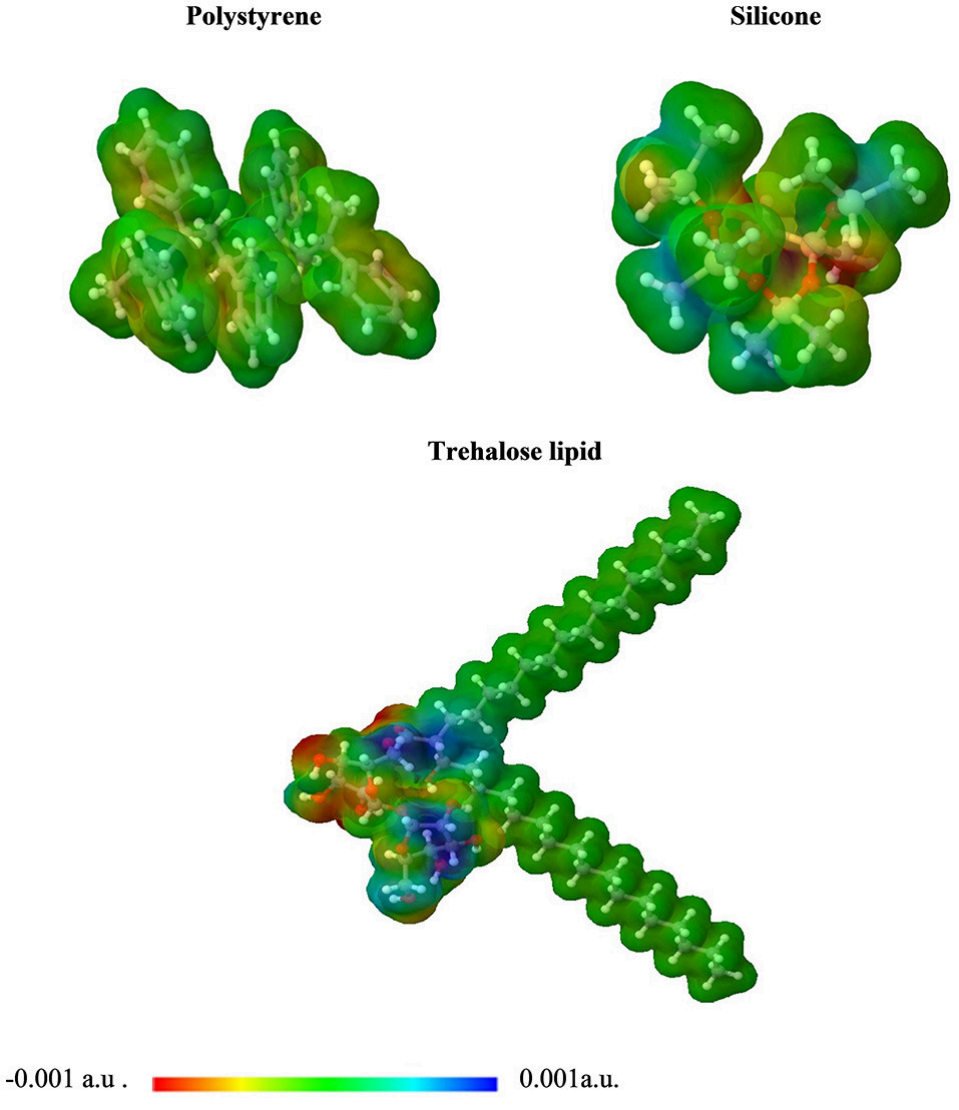
3D plot of the molecular electrostatic potential (MEP) surface for polystyrene, silicone, and trehalose lipid obtained at M06/6-31G(d,p).

Our intermolecular interaction energy calculations indicate that the trehalose lipid interacts strongly with the proposed surface fragments. The difference between the values of interaction energy estimated for silicone and polystyrene is about 3.6 kcal mol^−1^. The more stable complex with silicone is apparently due to the presence of relatively strong hydrogen bonds (1.71 Å) between the trehalose lipid and polysilicon molecule. In this case, the stabilizing effect arises from the interaction of permanent and induced multipole moments of molecules. The presence of benzene rings in the structure of polystyrene determines the nature of the interaction. In this case, the stabilizing effect results from the interactions of aromatic moieties responsible for the hydrophobic interacting environment. Besides, the estimated electrostatic potential surfaces of the investigated molecules allowed us to qualitatively predict the nature of the interactions (Figure 7). The data indicate that proton attack privileged fragments of trehalose lipid are localized mainly in its carbohydrates regions. Hence, one can expect that the formation of the stable complex could be possible in the negatively and positively charged parts of the molecules.

**Figure 8:**
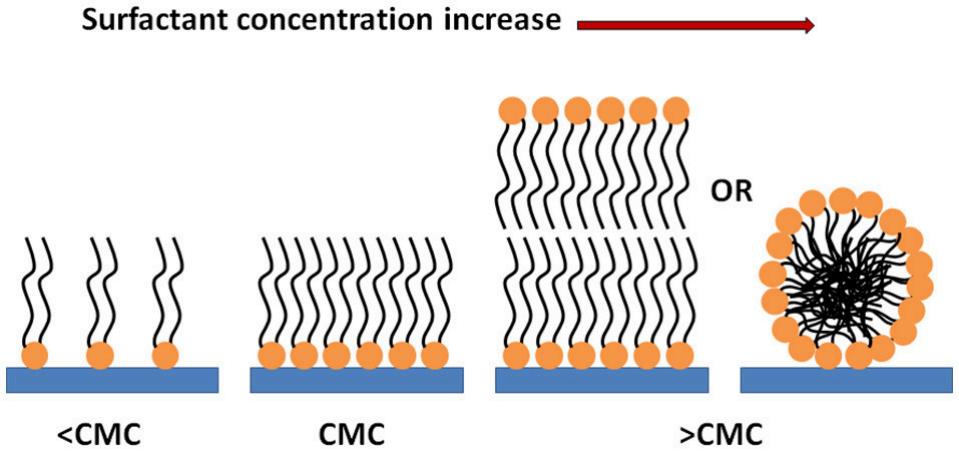
Scheme of the behavior of the trehalose lipid molecules in contact with the surfaces.

Based on our findings, we propose adsorption mechanisms of the trehalose lipid onto polystyrene and silicone surfaces; consisting of a monolayer adsorption mechanism when the trehalose lipid concentration is less than the CMC, and a bilayer or micelle adsorption mechanism when the concentration is greater than the CMC (Figure 8).

### Cellular toxicity

**Figure 9:**
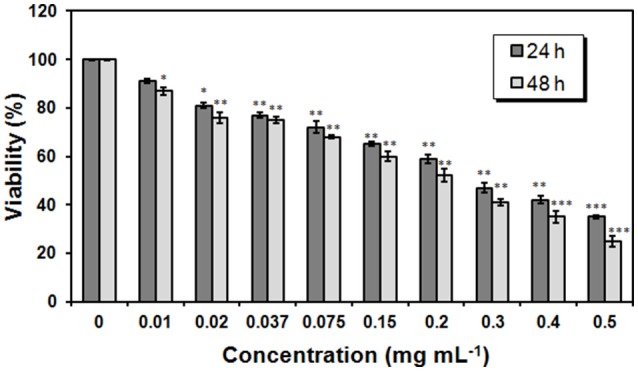
Proliferation rate of NHEK cells measured by MTT assay. NHEK cells were treated with 0-0.5 mg mL^−1^ concentrations of trehalose lipid for 24 h or 48 h. The bars represent the means ± SD of triplicate values for three independent experiments. ^*^0.05 > *P* >0.01, ^**^0.01 >*P* > 0.001, ^***^P < 0.001.

The proliferation rate of NHEK cells grown in the presence of trehalose lipid was measured by MTT assay. When the cells were treated with various concentrations of trehalose lipid for 24 and 48 h; we found that the cell survival rates were decreased in a dose- and time-dependent manner (Figure 9). After 24 h, cell viability decreased to values around 35% after exposure to the highest trehalose lipid concentration. As the time of incubation increases, the cell viability reduces and after 48 h of incubation it was observed that the viability reduced to 25% in case of 0.5 mg mL^−1^ (maximum concentration; Figure 9). The IC_50_ values of trehalose lipid on the NHEK cells for 24 and 48 h were 0.28 and 0.22 mg mL^−1^, respectively. The values of IC_50_ were higher than those obtained for keratinocytes with succinoyl bacterial trehalose lipid (IC_50_ was 0.09 mg mL^−1^). It proved that the trehalose lipid secreted by *R. fascians* BD8 is less irritating than biosurfactant produced by *Rhodococcus erythropolis* 51T7 (Marqués et al., 2009). Our results confirms the possible utility of this biosurfactant which acquire the safety standards for living organism.

## Conclusion

Here we show that the trehalose lipid synthesized by the BD8 strain of *R. fascians* is an attractive compound for the control and prevention of infection and might be employed in a diverse range of antimicrobial applications. The antimicrobial and antiadhesive properties of trehalose lipid against pathogenic bacteria and *C. albicans* adhesion, cell viability, and biofilm formation on silicone, polystyrene, and glass surfaces are reported. Up to 95% prevention of *C. albicans* adhesion to a polystyrene surface could be achieved by 0.5 mg mL^−1^ trehalose lipid. To the best of our knowledge, this work represents a first step toward the exploration of trehalose lipid interaction with medical surfaces using quantum chemical calculations. Our findings demonstrate the potential use of trehalose lipid in medical fields. Due to its surface tension properties, trehalose lipid can be used as a surface coating agent against microbial colonization of various surfaces (e.g., implants and urethral catheters).

## Author contributions

TJ, AK, and ML designed and supervised the study. TJ and ZC performed experiments, analyzed the data, and drafted the paper. All authors approved the submission and publication of all aspects of the work.

### Conflict of interest statement

The authors declare that the research was conducted in the absence of any commercial or financial relationships that could be construed as a potential conflict of interest.

## Supplementary material

Supplementary Movie 1 The most stable trehalose lipid - silicone complex.

Supplementary Movie 2 The most stable trehalose lipid - polystyrene complex.
