# Supplementary material for: Trehalose Lipid Biosurfactant Reduces Adhesion of Microbial Pathogens to Polystyrene and Silicone Surfaces: An Experimental and Computational Approach
Source: Front Microbiol. 2018 Oct 16;9:2441. doi: 10.3389/fmicb.2018.02441 (PMC6198247; doi:10.3389/fmicb.2018.02441)
Supplement: Supplementary file 3 [file Data_Sheet_1.docx]

Supplementary Material

Trehalose Lipid Biosurfactant Reduces Adhesion of Microbial Pathogens to Polystyrene and Silicone Surfaces: An Experimental and Computational Approach

**Tomasz Janek^1*^, Anna Krasowska^2^, Żaneta Czyżnikowska^1^ and Marcin Łukaszewicz^2*^**

^1^ Department of Inorganic Chemistry, Wroclaw Medical University, Wroclaw, Poland

^2^ Department of Biotransformation, Faculty of Biotechnology, University of Wroclaw, Wroclaw, Poland

*Correspondence:

Tomasz Janek

tomasz.w.janek@gmail.com, tomasz.janek@umed.wroc.pl

Marcin Łukaszewicz

marcin.lukaszewicz@uwr.edu.pl


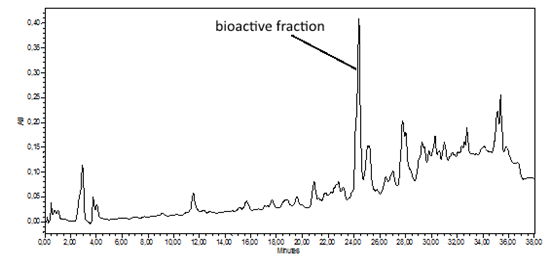


**Supplementary Figure S1.** RP-HPLC spectrogram of the surface-active compound with a retention time of 24.2 min obtained from *R. fascians* BD8. Absorbance was measured at 210 nm.


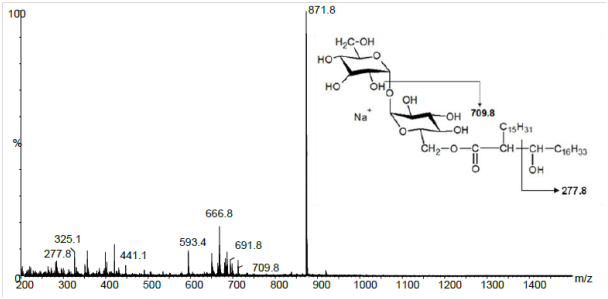


**Supplementary Figure S2.** MALDI-TOF mass spectrum of the purified trehalose lipid from *R. fascians* BD8.
